# Impact of Pulmonary Rehabilitation on Physical, Mental Health and Quality of Life in Children with Post-COVID-19 Condition: A 12-Month Quasi-Experimental Study

**DOI:** 10.3390/jcm15020535

**Published:** 2026-01-09

**Authors:** Aroia Goicoechea-Calvo, Natalia Navarro Expósito, Roser Coll-Fernández, Marc Colomer Giralt, Alberto Martín Saavedra, Alba González-Aumatell, María Méndez-Hernández, Clara Carreras-Abad, Mónica Moreira, Maria Giralt-López, Natàlia Pallarès, Cristian Tebe Cordomi, Agustí Rodríguez-Palmero, Carlos Rodrigo, Maria José Durà Mata

**Affiliations:** 1 Department of Rehabilitation, Germans Trias i Pujol University Hospital, 08916 Badalona, Spain; nnavarroe.germanstrias@gencat.cat (N.N.E.); rcollf.germanstrias@gencat.cat (R.C.-F.); mcolomer.germanstrias@gencat.cat (M.C.G.); amartins.germanstrias@gencat.cat (A.M.S.); mjdura.germanstrias@gencat.cat (M.J.D.M.); 2 Department of Pediatrics, Obstetrics and Gynecology, Preventive Medicine and Public Health, Faculty of Medicine, Universitat Autònoma de Barcelona, 08193 Cerdanyola del Vallès, Spain; agonzalezau.germanstrias@gencat.cat (A.G.-A.); mjmendez.germanstrias@gencat.cat (M.M.-H.); claracarrerasa.germanstrias@gencat.cat (C.C.-A.); arodriguezpalmero.germanstrias@gencat.cat (A.R.-P.); crodrigo.germanstrias@gencat.cat (C.R.); 3 Department of Pediatrics, Germans Trias i Pujol University Hospital, 08916 Badalona, Spain; 4 Germans Trias i Pujol Research Institute (IGTP), 08916 Badalona, Spain; 5 Child and Adolescent Psychiatry Department, Germans Trias I Pujol University Hospital, 08916 Badalona, Spain; mmoreiramar.germanstrias@gencat.cat (M.M.); mariagiralt.germanstrias@gencat.cat (M.G.-L.); 6 Psychiatry and Legal Medicine, Universitat Autònoma de Barcelona, 08193 Cerdanyola del Vallès, Spain; 7 Biostatistics Support and Research Unit, Germans Trias i Pujol Research Institute and Hospital (IGTP), 08916 Badalona, Spain; npallares@igtp.cat (N.P.); ctebe@igtp.cat (C.T.C.); 8 Department of Medicinet, Universitat Autònoma de Barcelona, 08193 Cerdanyola del Vallès, Spain

**Keywords:** paediatric post-COVID-19 condition, long COVID, rehabilitation, children, exercise capacity, physical activity, fatigue, young people, adolescents

## Abstract

**Background/Objectives:** Evidence on pulmonary rehabilitation (PR) in paediatric post-COVID-19 condition (PPCC) is scarce. This study aimed to evaluate the association of a PR programme with changes in physical and mental health and quality of life in PPCC over a 12-month follow-up. **Methods:** A quasi-experimental pre–post single-arm study was conducted, with no control group, in PPCC patients attending an outpatient PR unit. The primary outcome was change in exercise capacity (6 min walk test, 6MWT). Secondary outcomes included inspiratory and peripheral muscle strength, quadriceps muscle morphology by ultrasound, fatigue, physical activity, quality of life, and psychiatric symptoms, assessed using validated paediatric instruments. **Results:** A total of 115 PPCC patients (mean age 13.3 years; 66.1% female) completed the PR. 6MWD distance increased from 509 ± 87 to 546 ± 86 (+37 m; *p* < 0.001; D: 0.50). Handgrip strength increased by 2.4 kg, maximal inspiratory pressure increased by 15 cmH_2_O, physical activity increased by 2.4 points, fatigue score improved by 9.3 points, and quality of life improved by 11 points (all *p* < 0.001). Rectus femoris thickness increased by 0.56 mm (*p* = 0.005), psychiatric symptom scores decreased by 4.5 points (*p* < 0.001), and rectus femoris echo-intensity decreased (*p* = 0.003). **Conclusions:** Multidisciplinary PR appears feasible and potentially effective in improving physical function, psychological well-being, and quality of life in PPCC, supporting the need for evidence-based paediatric rehabilitation.

## 1. Introduction

It is increasingly recognised that a significant proportion of children and adolescents experience long-term symptoms after acute SARS-CoV-2 infection, collectively referred to as paediatric post-COVID-19 condition (PPCC). According to the World Health Organization (WHO), this condition is defined as the presence of symptoms lasting at least two months, which initially occur within three months of confirmed or probable SARS-CoV-2 infection, and have an impact on everyday functioning [1]. PPCC is characterised by a heterogeneous cluster of symptoms, including fatigue, respiratory manifestations, and cognitive and psychiatric symptoms, which can impair school performance, daily activities, and psychological well-being [2,3,4,5].

The pathophysiology of this condition remains poorly understood, and no specific curative treatment has been identified to date [6,7,8]. Among the proposed mechanisms, SARS-CoV-2-mediated vagus nerve dysfunction has been suggested as a contributor to persistent symptoms, given its central role in the regulation of involuntary visceral functions and the modulation of systemic inflammation [9,10,11]. In adults with post-COVID-19 condition (PCC, or Long COVID), inspiratory muscle weakness, particularly involving the diaphragm, has been increasingly postulated as a mechanism underlying persistent dyspnoea and exercise intolerance [12,13,14]. In addition, vagal pathways participate in the modulation of stress-related and emotional responses [15,16], which is of particular relevance in PPCC, as psychiatric manifestations constitute some of the most prevalent symptom clusters following systemic and respiratory symptoms [17].

Despite growing awareness of PPCC, evidence on the effectiveness of therapeutic exercise interventions in paediatric populations remains scarce. To date, no paediatric trials of pulmonary rehabilitation (PR) have been published, and most recommendations rely on extrapolation from adult studies or from other chronic paediatric diseases [18]. In adults with PCC, the majority of therapeutic exercise interventions have primarily focused on aerobic and strength training, sometimes incorporating breathing techniques, whereas studies including inspiratory muscle training remain limited [19]. Recent meta-analyses suggest that multicomponent PR programmes are associated with improvements in exercise capacity and health-related quality of life [20,21], although findings regarding fatigue and dyspnoea remain heterogeneous across studies [22,23].

Given this emerging evidence, PR constitutes a logical therapeutic approach for PPCC, combining aerobic reconditioning, strength training, and respiratory physiotherapy [24]. Clinically, dyspnoea, fatigue, exercise intolerance, and muscle weakness are among the most common symptoms prompting referral to rehabilitation in PPCC. These symptoms often worsen with physical activity, including low-intensity activities of daily living, and may be accompanied by post-exertional symptom exacerbation in a subset of patients, a pattern consistent with reduced exercise capacity. Beyond cardiovascular fitness alone [25], exercise capacity has been established as a powerful predictor of morbidity risk and early mortality across cardiovascular, metabolic, and respiratory diseases.

To address this gap, a dedicated multidisciplinary unit was implemented at the Germans Trias i Pujol University Hospital. In this context, we hypothesised that a structured PR programme combining aerobic, strength, and respiratory interventions could be associated with improvements in physical function and emotional well-being in PPCC. Therefore, the aim of this study was to evaluate the association of a PR programme with changes in exercise capacity (primary outcome), as well as fatigue, peripheral and inspiratory muscle strength, physical activity level, mental health symptoms, and health-related quality of life over a 12-month follow-up period in PPCC patients.

## 2. Materials and Methods

### 2.1. Study Design

This study used a quasi-experimental, single-arm pre–post design and was carried out within a specialised outpatient paediatric post-COVID-19 multidisciplinary unit at a tertiary referral hospital in Badalona (Barcelona, Spain). The study protocol was approved by the Institutional Review Board of the Germans Trias i Pujol University Hospital (PI-21-029; approved on 11 June 2021), and all procedures complied with the Declaration of Helsinki and relevant national and institutional regulations. Written informed consent was obtained from parents or legal guardians before enrolment. The clinical setting integrates expertise from rehabilitation medicine, physiotherapy, paediatrics, radiology, psychology, and psychiatry. Clinical evaluation and follow-up of children and adolescents with suspected PPCC were initially performed as part of routine clinical care within the newly established unit. Referrals originated from primary care practices, secondary hospitals within the regional health network, the adult PCC unit of the same institution, and the Catalan patient advocacy organisation. Participants fulfilling the inclusion criteria were enrolled consecutively. The study was conducted between 11 June 2021 and 22 November 2023. Reporting of the study adhered to the STROBE guidelines for observational research.

### 2.2. Participants and Setting

The study population consisted of children and adolescents between 8 and 17 years of age who were evaluated at our multidisciplinary unit following referral. Participants were previously described in earlier cross-sectional publications from our group [26,27]. In the present work, all consecutive PPCC patients who completed the PR programme and met the inclusion criteria were included in this pre–post analysis. Participants were eligible if they met all the following conditions: they reported at least three persistent symptoms consistent with PPCC lasting more than 12 weeks after acute SARS-CoV-2 infection, irrespective of prior hospital admission; symptoms had not been present before the infection and were associated with functional limitations in daily life; and SARS-CoV-2 infection had been microbiologically confirmed or was considered highly probable based on compatible clinical presentation in the context of household exposure during periods of community transmission when diagnostic testing was not readily available. Symptoms considered compatible with PPCC included, but were not limited to, fatigue, dyspnoea, cognitive complaints, musculoskeletal pain, muscle weakness, sleep disturbances, headache, palpitations, chest discomfort or oppression, orthostatic intolerance, sensory disturbances (including anosmia, ageusia or dysgeusia, tinnitus, hearing loss, photophobia and phonophobia), gastrointestinal symptoms (such as abdominal pain, diarrhoea, nausea, vomiting or dyspepsia), dizziness, cough, chills or persistent fever, cutaneous manifestations, swallowing difficulties, voice changes, and nasal symptoms. Participants were excluded if they or their legal guardians were unable to provide informed consent, if follow-up assessments were not attended, or if pre-existing psychiatric, cognitive, neurological, or musculoskeletal disorders interfered with the ability to complete the study procedures or perform physical evaluations. In addition, participants who attended fewer than 50% of the supervised PR sessions were withdrawn from the programme and excluded from the final analysis, in accordance with routine clinical practice in our unit.

### 2.3. Study Procedures and Data Collection

Clinical information was extracted and reviewed by the study team from participants’ records within the unit. Data were gathered during routine outpatient visits and were documented using structured forms embedded in the hospital’s electronic record system. Information was obtained through anamnesis, physical examination, and validated questionnaires assessing fatigue, physical activity, quality of life, and mental health. The dataset comprised demographic characteristics, personal and family medical background, information related to SARS-CoV-2 infection, clinical features during the acute phase of COVID-19, and ongoing symptoms persisting after the infection. Additional data included laboratory results (such as haematological and biochemical profiles, nutritional markers, antinuclear antibodies, thyroid function tests, and SARS-CoV-2 serology), findings from the physical examination, anthropometric status (body mass index, derived from measured weight and height), and physiological parameters including heart rate, blood pressure, respiratory rate, and peripheral oxygen saturation. Instrumental investigations such as electrocardiography, spirometry, lung ultrasonography, and chest radiography were also collected. In participants without virological or serological confirmation of SARS-CoV-2 infection (PCR, antigen testing, or serology), cellular immune responses were evaluated using a SARS-CoV-2–specific T-cell functional assay. To ensure the secure processing of personal data, appropriate technical and organisational measures were applied so that, by default, only the information strictly necessary for each specific purpose was processed. The principal investigators were responsible for entering data into a purpose-built, coded database devoid of personally identifiable information. Each enrolled participant was assigned a unique study code, which was used for all data entry and storage within a secure, password-protected institutional server with restricted access. A designated member of the research team supervised the collection and management of data related to the main outcome variables. All data handling procedures were conducted in accordance with institutional data protection policies and the European General Data Protection Regulation (EU 2016/679).

#### 2.3.1. Physical Function Assessments

The 6 min walk test (6MWT) [28] was used to evaluate exercise capacity. Participants were asked to walk back and forth along a flat, straight, hard-surfaced corridor at the fastest pace they could comfortably sustain for six minutes. The walking course was clearly marked by two cones 24 metres apart, and the total distance covered in metres was considered the primary outcome. Perceived breathlessness and exertional fatigue were quantified using the Borg scale ranging from 0 (no symptoms) to 10 (maximal symptom intensity) [29]. Ratings were obtained immediately before starting the walking test and again upon its completion.

Handgrip strength (HGS) was assessed bilaterally using a hand dynamometer (Jamar^®^ Hydraulic Hand Dynamometer, Performance Health Supply, LLC, Cedarburg, WI, USA) in accordance with a standardised testing procedure [30]. Three maximal contractions were performed for each hand, and the highest recorded value was retained and expressed in kilograms as the absolute HGS. Hand dominance was documented for each participant. Measured values were subsequently normalised by comparison with age-, sex-, and dominance-specific reference data derived from the Spanish paediatric population [31], and results were expressed as a percentage of the predicted value. Given the known influence of sex, age, and dominance on handgrip performance, relative values (% predicted) were used as the primary indicator for analytical purposes [32].

Inspiratory muscle strength was assessed by measuring maximal inspiratory pressure (PImax) using a digital mouth pressure device (MicroRPM; MicroMedical GmbH, Hoechberg, Germany), following the recommendations of the American Thoracic Society and European Respiratory Society (ATS/ERS) [33]. Measurements were performed with participants seated upright, with the trunk supported, hips and knees flexed at 90 degrees, and both feet resting on the floor. Before formal assessment, two practice manoeuvres were completed to familiarise participants with the procedure. A nasal clip was applied for all measurements. Participants were instructed to generate a maximal inspiratory effort starting from residual lung volume, and pressure values were recorded. Three technically acceptable and reproducible manoeuvres were obtained, defined by a variability of less than 10% between attempts, and the highest value was retained for analysis. A one-minute rest period was allowed between successive trials. Age- and sex-specific paediatric reference values reported by Szeinberg et al. [34], expressed in cmH_2_O, were used to define the lower limit of normal (LLN). Each participant’s PImax was categorized as below LLN, within the normal range, or not evaluable. Due to logistical constraints during the initial phase of unit implementation coinciding with the COVID-19 pandemic, PImax measurements could not be systematically performed in all participants.

Ultrasound imaging was performed using a portable system (VINNO 5; VINNO Technology Co., Ltd., Suzhou, China) to acquire transverse B-mode images with a linear transducer of the rectus femoris (RF). Ultrasound settings were standardised across all participants, with a 7.5 MHz frequency, a depth of 7.0 cm, and a gain of 20 dB. All examinations were performed by a single operator, an experienced rehabilitation physician trained in rehabilitative ultrasound imaging, who underwent supervised practice and mentoring prior to data collection. For image acquisition, participants were positioned in a supine posture, with the hips placed in a neutral alignment and both knees fully extended and relaxed. The anatomical landmark was located at the distal third of the line connecting the anterosuperior iliac spine (ASIS) and the superior border of the patella (SBP), on both limbs, with limb dominance recorded [35]. The distal third site (66.6% of the total distance) was marked with a skin pen. A layer of ultrasound gel was used to optimise acoustic coupling, and the transducer was maintained perpendicular to the skin with minimal contact force to limit tissue compression. The measurements obtained included rectus femoris muscle thickness (RF MT) and rectus femoris echo-intensity (RF EI). Muscle thickness of rectus femoris (RF MT) was defined as the distance, in millimetres, between the superficial and deep fascia of the rectus femoris muscle. Two consecutive measurements were obtained at each site, and their average value was used for subsequent analyses. The reproducibility of muscle thickness measurements was evaluated by calculating the intra-class correlation coefficient (ICC) with 95% confidence intervals, based on two repeated measurements performed by the same examiner. RF EI in the dominant limb was categorised using the semi-quantitative Heckmatt scale of muscle echogenicity [36]: Grade I indicated normal muscle appearance; Grade II corresponded to increased muscle echogenicity with preserved bone reflection; Grade III reflected a further increase in muscle echogenicity with decreased bone reflection; and Grade IV represented markedly increased muscle echogenicity with absent bone reflection.

#### 2.3.2. Physical Activity and Fatigue Assessments

Physical activity was measured using the Assessment of Physical Activity Levels Questionnaire (APALQ) for children and adolescents [37,38]. The instrument yield scores between 5 and 22, with higher values corresponding to higher levels of habitual physical activity. Based on established cut-offs, participants were classified as sedentary (scores ≤ 10), moderately active (scores 11–16), or very active (scores ≥ 17).

Perceived fatigue was assessed with the Pediatric Functional Assessment of Chronic Illness Therapy—Fatigue (Peds FACIT-F), a 13-item self-report questionnaire developed for use in paediatric populations aged 8 to 18 years [39,40,41]. The scale captures both fatigue intensity and its impact on everyday functioning. Summary scores range from 0 to 52, where higher values indicate lower levels of fatigue. Fatigue severity was categorised into five levels: fatigue-free (≥45), low fatigue (31–44), moderate fatigue (21–30), high fatigue (11–20), and very high fatigue (0–10).

#### 2.3.3. Quality of Life Assessments

Health-related quality of life was assessed using the Pediatric Quality of Life Inventory (PedsQL), a self-reported questionnaire designed for children and their parents, which includes a generic core module comprising three domains: Physical Health, Psychosocial Health, and Global Health. Items were scored on a five-point response scale ranging from 0 (never) to 4 (almost always), with higher scores representing better health-related quality of life [42].

#### 2.3.4. Mental Health Assessments

Mental health was evaluated using the Spanish-language version of the Pediatric Symptom Checklist (PSC), a validated screening tool for detecting emotional and behavioural problems in children and adolescents. The questionnaire comprises 35 items rated on a three-point scale (0 = never, 1 = sometimes, 2 = often). For participants under 11 years, the PSC was completed by parents or relatives, whereas those aged 11–17 years self-administered the Youth PSC. Total scores were calculated, and participants scoring above the established cut-offs (≥28 for PSC; ≥30 for Youth PSC) were referred for further evaluation by a mental health professional [43].

##### Mental Health Evaluations

All first-time evaluations conducted in our service comprised a comprehensive diagnostic assessment and a structured psychoeducational intervention addressing emotional and behavioural symptomatology. Psychoeducation included information on the bidirectional mind–body relationship, with particular emphasis on the impact of emotional processes on the onset, maintenance, and clinical course of somatic conditions. Follow-up assessments were scheduled according to clinical need and were conducted by psychiatrists or clinical psychologists within the child and adolescent liaison psychiatry service at our centre. When clinically indicated, pharmacological treatment was initiated in accordance with standard clinical criteria, most commonly involving selective serotonin reuptake inhibitors for depressive and/or anxiety symptomatology, as well as hypnotic agents, given the high prevalence of sleep disturbances in this patient population and their significant impact on overall health status. For patients already engaged with the mental health care network, continuity of treatment with their usual clinicians was prioritised. This approach reflects routine clinical practice and provides an ecologically valid representation of the context in which assessments were conducted.

#### 2.3.5. School and Activity Participation

Data on school attendance and engagement in sport or physical activity were obtained through a self-reported questionnaire. The questionnaire included the following variables: regular engagement in sport or physical activity prior to COVID-19 (yes/no); ability to return to the same sport or physical activity after COVID-19 (yes/no); self-reported difficulty engaging in physical activity at Pre-RHB (yes/no); the number of hours per week spent in sport or physical activity prior to COVID-19 (0, 0–2, 2–4, 4–6, >6), prior to PR, and post-PR. School attendance was categorised as regular, partial, or non-attendance.

#### 2.3.6. Pulmonary Rehabilitation Programme

Prior to starting the PR, all patients underwent comprehensive medical evaluation, including physical examination and routine physiological measurements, such as blood pressure, heart rate, respiratory rate, and peripheral oxygen saturation. Patients with contraindicating conditions (e.g., musculoskeletal, neurological, or cardiopulmonary impairments) were excluded. Training sessions were temporarily suspended in the presence of fever (>37.5 °C), resting heart rate exceeding 100 beats per minute, systolic blood pressure above 160 mmHg or below 85 mmHg, or peripheral oxygen saturation below 85%, or symptoms such as dizziness or vomiting. The PR followed a multidisciplinary, individualised approach tailored physical and respiratory therapy, patient and family education, promotion of healthy lifestyle habits, and psychological and social support. Educational components focused on the safe resumption of daily activities and activity pacing according to individual symptom tolerance. The PR lasted 5 weeks and consisted of ten supervised sessions (2 sessions per week) complemented by a structured home-based exercise programme on the remaining days. Attendance to supervised sessions was monitored as part of routine clinical care, and a minimum attendance threshold was required to ensure adequate exposure to the intervention. For patients unable to attend in person due to logistical reasons, a tele-rehabilitation option was offered. In both in-person and tele-rehabilitation modalities, the structure, content, and weekly exercise volume were equivalent. Across the programme in both modalities, and including the home-based exercise component, participants were prescribed the following weekly distribution of exercise: Aerobic training: 5 days per week; Respiratory physiotherapy: 5 days per week. Strength training: 3 days per week. Proprioception and balance training: 3 days per week. Each supervised session lasted approximately 60 min and included the following components: Warm-up and joint mobilisation/flexibility exercises (5 min); Aerobic training (treadmill or stationary cycle): starting 10 min, and progressively increasing toward 20 min by week 4, at moderate intensity corresponding to 4–6 on the Borg perceived exertion scale (0–10 scale). Respiratory physiotherapy in seated position (15 min), comprising: (1) Ventilatory control exercises, involving deep nasal inhalation and slow exhalation through pursed lips, upper thoracic and lateral costal breathing, and abdomino-diaphragmatic breathing. (2) Inspiratory muscle training, initial load at 30% of MIP, with incremental increases weekly (≥2 cm H_2_O) as tolerated. The IMT protocol consisted of 1 set of 10 repetitions, performed twice daily (morning/afternoon) during the first week. In week 2, the IMT progressed to 2 sets of 10 repetitions twice daily. From week 3 onwards, the number of sets was gradually increased according to individual tolerance, up to a maximum of 5 sets of 10 repetitions twice daily. During supervised sessions, patients were guided to exercise at a moderate intensity corresponding to 4–6 on the Borg scale. Load and volume were adjusted based on serial PImax assessments every 1–2 weeks, according to individual tolerance and progression. (3) Energy conservation and dyspnoea management education; Strength training (10 min) using elastic resistance bands and bodyweight functional movements (e.g., squats, arm raises, heel raises). Training intensity was maintained at a moderate level (Borg scale 4–6) and progressively increased by adjusting band load, repetitions, or movement complexity. Emphasis was placed on large muscle groups to enhance overall functional capacity and support activities of daily living. Rest intervals of 30–60 s were allowed between sets, ensuring adequate recovery and prevention of post-exertional symptom exacerbation. Proprioception exercises (from week 3 onwards; 5 min) focusing on coordination and balance on unstable surfaces; Cool-down (5 min) including low-intensity respiratory exercises, static stretching, and joint mobilization to gradually reduce heart rate. For participants experiencing post-exertional symptom exacerbation, rehabilitation strategies prioritised education on conservative resumption of everyday activities, tailored pacing, and symptom-guided activity management. Graded exercise was avoided and patients were instructed to avoid exercising to the point of fatigue or symptom worsening, both during activity and in the subsequent hours or days. When symptom exacerbation occurred, patients were advised to stop, rest and pace activities. Additional guidance included heart rate monitoring and energy conservation strategies to support self-management and minimise the risk of symptom flares.

Assessment time points were defined as Pre-RHB (immediately before rehabilitation), Post-RHB (immediately after rehabilitation), 6M follow-up (6 months after rehabilitation), and 12M follow-up (12 months after rehabilitation).

##### Home-Based Exercise Programme

In addition to the supervised sessions, a structured home-based exercise programme was prescribed on the remaining days of the week. Each home-based session included a 5 min warm-up (joint mobilisation and flexibility exercises) and a 5 min cool-down (stretching and mobility exercises), as previously instructed during supervised sessions. Aerobic training was prescribed at moderate intensity and consisted of stationary or outdoor cycling, elliptical training, light jogging, or swimming, depending on individual preference and tolerance (approximately 30 min per session, performed continuously or in bouts of at least 10 min). Walking was recommended as the basic form of physical activity and encouraged on most days of the week (approximately 30 min per day, continuously or in bouts of at least 10 min). Respiratory physiotherapy was performed for approximately 15 min and included ventilatory control exercises (thoracic mobility, lateral costal breathing, abdomino-diaphragmatic breathing, and pursed-lip exhalation). In participants with reduced PImax, IMT using a threshold device was prescribed following the same progression principles as during supervised sessions. Strength training using elastic resistance bands and functional bodyweight exercises targeting major muscle groups, as well as proprioceptive and balance exercises (from week 3 onwards), were incorporated into the home-based programme. Exercise intensity across all components was guided by perceived exertion (Borg scale 4–6), provided it was well tolerated and did not induce symptom exacerbation.

### 2.4. Statistical Analysis

This study analysed PPCC patients who had been previously characterised in a cross-sectional comparison with healthy controls. All consecutive PPCC patients who subsequently participated in the PR and completed both baseline and post-rehabilitation assessments were included. Therefore, the sample size (nN = 115) was determined by clinical availability rather than by a formal a priori power calculation. Our prior data [26] indicate that the paired differences in the 6MWT are approximately normally distributed with a standard deviation of differences around 80–90 m. Under these assumptions, and with 80% power (two-sided α = 0.05), the available sample would allow detection of a mean paired difference of approximately 22–25 m. These differences are considered clinically relevant, so the number of subjects available at baseline was considered adequate to conduct an exploratory pre–post study. Baseline characteristics are presented using descriptive statistics (Table 1). Categorical variables are described using frequencies and percentages of each category, while continuous variables are summarised as mean values with corresponding standard deviations or median and interquartile range (depending on variable distribution assessed with quantile-quantile plot). Differences in outcome variables Pre-RHB and Post-RHB were analysed by calculating the mean difference or the pseudo-median, based on variable distribution, with its 95% confidence interval and the Cohen’s D with its 95% confidence interval. Comparisons were performed with paired *t* tests or paired Wilcoxon signed-rank test, according to variable distribution, and McNemar’s tests for categorical variables. To analyse the evolution of main continuous outcomes across all time points (Pre-RHB, Post-RHB, 6M follow-up, and 12M follow-up) mean and standard errors were calculated at each time point and represented graphically. Data available for each outcome at each time point was also reported. Linear mixed models were used to assess outcome trend across the four time points. Given the exploratory nature of the study, analyses were interpreted descriptively, and no formal adjustment for multiplicity was applied. Missing data were not imputed; instead, all analyses were conducted using available data, and the number of paired observations contributing to each pre-post comparison is reported (see the “Pairs” column in Table 2). Statistical analysis was reported in accordance with the STROBE guidelines [44].

All statistical analyses were carried out using R software (version 4.4.0, released 14 June 2024) running under Windows.

## 3. Results

### 3.1. Characteristics of the Study Sample

**Figure 1:**
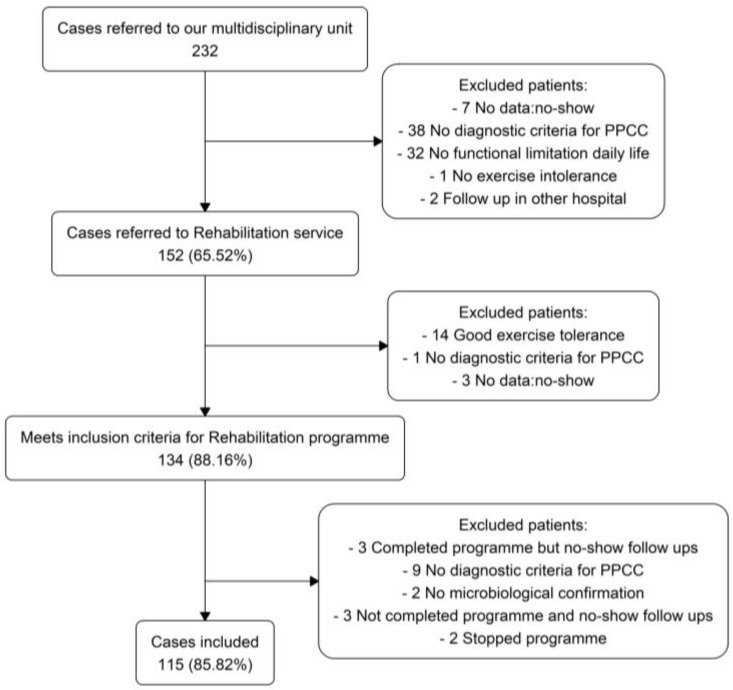
Flow diagram of study inclusion.

Figure 1 illustrates the participant selection process. Among the 232 patients initially referred to our multidisciplinary unit, 152 (65.52%) were subsequently referred to the rehabilitation service. After screening by the rehabilitation team, 134 patients (88.16% of those referred) were deemed eligible to initiate a PR programme based on the presence of persistent functional limitations in daily life and/or exercise intolerance, even in cases with initially uncertain or not yet fully defined clinical features of PPCC. Of the 134 patients deemed eligible after screening, 19 were excluded from the final analyses during the period between Pre-RHB and Post-RHB, for the following reasons: three completed the programme but did not attend Post-RHB follow-up assessments; nine did not fulfil the unit’s predefined diagnostic criteria for PPCC; two lacked microbiological confirmation of SARS-CoV-2 infection; three did not complete the programme due to attendance below the predefined minimum threshold (50% of supervised sessions); and two discontinued the programme voluntarily. The final study sample therefore comprised 115 children and adolescents (85.82% of those eligible after screening) who completed the programme and were included in the final analysis.

The characteristics of the study population are detailed in Table 1. Participants were on average 13.31 ± 2.25 years old (range 8–17), and females accounted for the 66.09% of the sample. Thirty-eight percent had no pre-existing medical conditions (detailed in Supplementary Table S1). A positive diagnostic test for SARS-CoV-2 was documented in 98.26% of participants, most commonly by PCR (54.39%). Two patients (1.75%) did not have microbiological confirmation because their infection occurred at the very beginning of the pandemic, when diagnostic testing was not yet available; however, both had a clinical presentation and exposure history compatible with SARS-CoV-2 infection, as specified in the inclusion criteria. Regarding weight status, 81.74% of the participants fell within the healthy range, whereas 6.96% were overweight and 11.30% met criteria for obesity.

Prior to COVID-19 infection, 80.87% of our study sample reported participation in sport or physical activity, with a median of 3.0 (1.5–6) hours per week. Following SARS-CoV-2 infection, 62.37% did not resume their pre-infection levels of sport or physical activity, while 37.63% reported having resumed the activities they engaged in before the infection, and all PPCC patients reported difficulty engaging in physical activity. At Pre-RHB, median weekly physical activity decreased to 0 (0–1.5) hours, whereas at Post-RHB it was 2 (0–3) hours. Regarding school attendance, at Pre-RHB, 43.48% of participants had reduced school attendance, with 31.30% attending school part-time and 12.17% not attending school at all. At Post-RHB, regular attendance was higher (71.30%) compared with Pre-RHB (56.52%), with a corresponding reduction in partial or non-attendance.

**Table 1:** Baseline characteristics of the study population (N = 115).

Variables	PPCC Patients
Sex	
Female	76 (66.09)
Male	39 (33.91)
Age, *n* (years)	13.31 ± 2.25
Microbiological confirmation SARS-CoV-2 infection	
No ^b^	2 (1.74)
Yes	113 (98.26)
Diagnostic method	
RT-qPCR	62 (54.39)
Rapid antigen detection test	33 (28.95)
Serological test ^c^	9 (7.89)
Cellular immunity ^d^	8 (7.02)
No microbiological/immunological confirmation	2 (1.75)
Background medical conditions	
No	38 (33.04)
Yes	77 (66.96)
BMI, kg/m^2^	21.33 ± 4.50
Weight percentile	51.70 ± 31.4
Healthy weight by percentile	94 (81.74)
Overweight by percentile (>90)	8 (6.96)
Obesity by percentile (>97)	13 (11.30)
Regular engagement in sport or physical activity prior to COVID-19	
No	22 (19.13)
Yes	93 (80.87)
Hours per week spent in sport or physical activity prior to COVID-19	3.00 (1.5; 6.0)
0 h	21 (18.26)
0–2 h	24 (20.87)
2–4 h	30 (26.09)
4–6 h	16 (13.91)
>6 h	24 (20.87)
Ability to return to the same sport or physical activity after COVID-19	
No	58 (62.37)
Yes	35 (37.63)
Difficulty engaging in physical activity Pre-RHB	
No	0 (0.00)
Yes	115 (0.00)
Hours per week spent in sport or physical activity Pre-RHB	0 (0.0; 1.5)
Hours per week spent in sport or physical activity Post-RHB	2 (0.0; 3.0)
School attendance Pre-RHB	
Regular attendance	65 (56.52)
Partial attendance	36 (31.30)
Non-attendance	14 (12.17)
School attendance Post-RHB	
Regular attendance	82 (71.30)
Partial attendance	21 (18.26)
Non-attendance	12 (10.43)

Abbreviations: SARS-CoV-2, severe acute respiratory syndrome coronavirus 2; BMI, body mass index; PPCC, paediatric post-COVID-19 condition; COVID-19, coronavirus disease 2019; RT-qPCR, real-time quantitative reverse transcription polymerase chain reaction; Pre-RHB, immediately before rehabilitation; Post-RHB, immediately after rehabilitation. Notes: ^b^ no microbiological or immunological confirmation: compatible COVID-19 symptoms in participants with documented household exposure to a confirmed SARS-CoV-2 case during periods of community transmission when diagnostic testing was not available. ^c^ serological test: SARS-CoV-2 spike-binding neutralizing antibody by using chemiluminescent immunoassays. ^d^ cellular immunity: Surface activation-induced markers (AIM) assay. Data are presented as the mean ± SD, median (Q1; Q3) or frequency (percentage). Post-RHB values for physical activity and school attendance are provided for descriptive purposes only; statistical comparisons are shown in Table 2.

### 3.2. Clinical Characteristics

Because follow-up assessments varied by outcome and time point, the number of participants contributing paired data differed across variables; paired sample sizes are reported in Table 2 (column “Pairs”), and sample sizes at each assessment time point are detailed in Supplementary Tables S2–S9.

**Figure 2:**
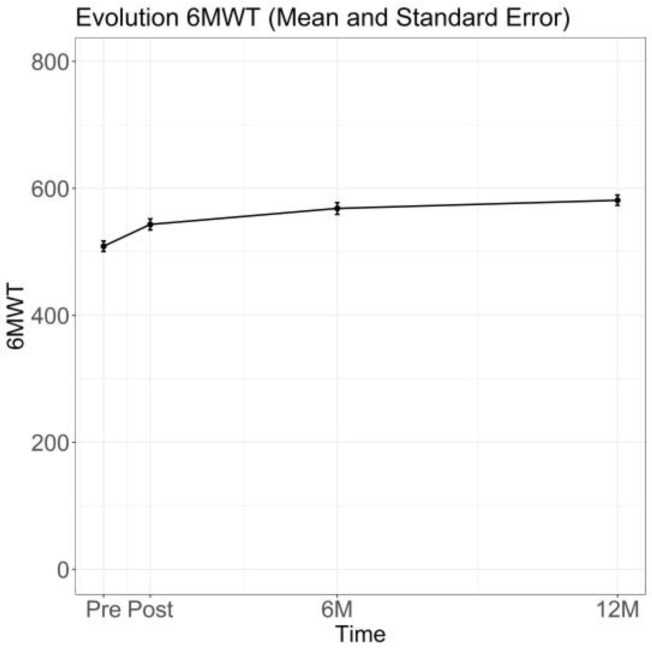
Evolution of 6MWT Performance Over Time. Values are shown as mean ± standard error at each assessment time point. Sample sizes at each time point are reported in Supplementary Table S2.

Analysis of physical function variables (Table 2) showed significant changes in exercise capacity, the primary outcome, from Pre-RHB to Post-RHB. Mean 6MWT distance increased from 509 ± 87 m at Pre-RHB to 546 ± 86 m at Post-RHB (mean difference = 37 m, 95% CI 23–52; *p* < 0.001), with a moderate effect size (Cohen’s d = 0.50). In parallel, dyspnoea and fatigue perception during the test decreased. Borg dyspnoea scores measured before the 6MWT showed a non-significant change (*p* = 0.083; Cohen’s d = −0.19), whereas post-test scores declined significantly from 5.00 to 3.00 (mean difference = 1.5, 95% CI 1.00–2.00; *p* < 0.001; Cohen’s d = −0.40). Fatigue scores decreased both before the test (2.00 to 0.00; mean difference = 2.00, 95% CI 0.50–2.50; *p* < 0.001; Cohen’s d = −0.38) and after the test (6.00 to 4.00 (mean difference = 2.00, 95% CI 1.00–2.5; *p* < 0.001; Cohen’s d = −0.48). Follow-up assessments (Figure 2) showed that these changes in exercise capacity were sustained, with 6MWT distance continuing to rise slightly and reaching mean values of approximately 580–590 m at the 12M follow-up.

**Table 2:** Comparison of Pre-RHB and Post-RHB physical function outcomes, fatigue, mental health and quality of life outcomes.

Variables	Pairs	Pre-RHB	Post-RHB	Difference [95%CI]	*p* Value	Cohen’s d [95% CI]
6MWT, m	103	509.22 ± 87.38	546.47 ± 86.38	37.00 [23.00; 52.00]	<0.001	0.50 [0.29; 0.70]
Dyspnoea (Borg 0–10), before 6MWT	103	0.00 (0.00, 2.00)	0.00 (0.00, 2.00)	1.00 [0.00; 2.00]	0.083	−0.19 [−0.38; 0.01]
Dyspnoea (Borg 0–10), after 6MWT	103	5.00 (2.00, 6.00)	3.00 (2.00, 5.00)	1.50 [1.00; 2.00]	<0.001	−0.40 [−0.60; −0.20]
Fatigue (Borg 0–10), before 6MWT	103	2.00 (0.00, 5.00)	0.00 (0.00, 3.00)	2.00 [0.50; 2.50]	<0.001	−0.38 [−0.58; −0.18]
Fatigue (Borg 0–10), after 6MWT	103	6.00 (3.00, 7.00)	4.00 (2.00, 6.00)	2.00 [1.00; 2.50]	<0.001	−0.48 [−0.68; −0.27]
BMI, kg/m^2^	104	21.39 ± 4.63	21.38 ± 4.60	−0.01 [−0.21; 0.19]	0.92	−0.01 [−0.20; 0.18]
HGS (D), kg	107	20.36 ± 8.72	22.77 ± 9.57	2.40 [1.40; 3.40]	<0.001	0.46 [0.26; 0.66]
HGS, % predicted (D)	107	82.63 ± 28.39	90.80 ± 30.81	8.20 [4.60; 12.00]	<0.001	0.44 [0.24; 0.64]
HGS (ND), kg	107	19.03 ± 8.60	20.73 ± 8.91	1.70 [0.68; 2.70]	0.001	0.32 [0.12; 0.51]
HGS, % predicted (ND)	107	81.67 ± 30.20	87.84 ± 30.91	6.20 [2.30; 10.00]	0.002	0.30 [0.11; 0.50]
PImax, cmH_2_O	57	70.46 ± 25.98	85.84 ± 29.27	15.00 [11.00; 20.00]	<0.001	0.92 [0.60; 1.20]
Reference LLN PImax (Szeinberg), cmH_2_O	57	95.60 ± 11.68	94.86 ± 11.08	−0.74 [−1.80; 0.34]	0.177	−0.18 [−0.44; 0.08]
Szeinberg Limit	60					
Below LLN by Szeinberg		44 (73.33)	29 (48.33)			
Within normal range by Szeinberg		7 (11.67)	23 (38.33)			
Not evaluable by Szeinberg		9 (15.00)	8 (13.33)			
RF MT (D), mm	96	12.88 ± 3.71	13.43 ± 3.75	0.56 [0.18; 0.94]	0.005	0.30 [0.09; 0.50]
RF MT (ND), mm	96	12.25 ± 3.49	13.12 ± 3.59	0.87 [0.50; 1.20]	<0.001	0.47 [0.26; 0.68]
RF EI	96				0.003	
RF EI I		76 (79.17)	87 (90.63)			
RF EI II/III/IV		20 (20.83)	9 (9.38)			
APALQ	115	7.94 ± 3.14	10.37 ± 3.50	2.40 [1.80; 3.00]	<0.001	0.77 [0.56; 0.97]
APALQ categories	115					
Sedentary (5–10)		89 (77.39)	57 (49.57)			
Moderately active (11–16)		24 (20.87)	54 (46.96)			
Very active (>17)		2 (1.74)	4 (3.48)			
Peds FACIT-F	110	25.00 ± 10.94	34.28 ± 12.48	9.30 [7.10; 11.00]	<0.001	0.80 [0.58; 1.00]
Peds FACIT-F categories	110					
Fatigue-free (45–52 score)		5 (4.55)	29 (26.36)			
Low (31–44 score)		34 (30.91)	42 (38.18)			
Moderate (21–30 score)		30 (27.27)	21 (19.09)			
High (11–20 score)		32 (29.09)	14 (12.73)			
Very high (0–10 score)		9 (8.18)	4 (3.64)			
PSC	94	23.50 ± 9.18	18.99 ± 9.05	−4.50 [−6.20; −2.90]	<0.001	−0.56 [−0.77; −0.34]
PSC categories	96					
Positive		24 (25.00)	15 (15.62)			
Negative		72 (75.00)	81 (84.38)			
PedsQL categories						
Physical Health	94	42.56 ± 21.11	58.43 ± 23.39	16.00 [11.00; 21.00]	<0.001	0.69 [0.47; 0.92]
Psychosocial Health	94	58.67 ± 17.43	64.85 ± 18.29	6.20 [2.60; 9.70]	<0.001	0.36 [0.15; 0.57]
Global Health	89	50.40 ± 17.87	61.44 ± 19.44	11.00 [7.10; 15.00]	<0.001	0.58 [0.36; 0.81]

Abbreviations: Pre-RHB, immediately before rehabilitation; Post-RHB, immediately after rehabilitation; BMI, body mass index; APALQ, Assessment of Physical Activity Levels Questionnaire; Peds FACIT-F, Pediatric Functional Assessment of Chronic Illness Therapy-Fatigue; 6MWT, 6 min walk test; before 6MWT, immediately before starting the 6MWT; after 6MWT, immediately after completing the 6MWT; PSC, Pediatric Symptom Checklist; PedsQL, Pediatric Quality of Life Inventory; PImax, maximal inspiratory pressure; LLN, lower limit of normal (reference values in cmH_2_O according to age and sex from Szeinberg et al. [34]); RF MT, rectus femoris muscle thickness; RF EI, rectus femoris echo-intensity, classified using a four-grade Heckmatt scale [36] (Grade I: normal muscle appearance; Grade II: increased echogenicity with preserved bone reflection; Grade III: further increased echogenicity with reduced bone reflection; Grade IV: markedly increased echogenicity with absent bone reflection); Borg, Borg scale (0–10) [29]; HGS, handgrip strength; D, dominant; ND, non-dominant; PPCC, paediatric post-COVID-19 condition; 95% CI, 95% confidence interval. Notes: Data are presented as the mean ± SD, median (Q1; Q3) or frequency (percentage). Differences and 95% CIs were calculated using pseudo-medians (dyspnoea and fatigue, Borg scale) or mean differences (all other continuous variables). Cohen’s d and 95% CI were used to report effect size. *p*-values were calculated using paired Wilcoxon signed-rank test (dyspnoea and fatigue, Borg scale), McNemar’s test (RF EI categories) and paired *t*-test (all other variables). Sample sizes for paired analyses vary by outcome and are reported in the “Pairs” column. Classifications “below LLN”, “within normal range”, and “not evaluable” were based on these reference values reported by Szeinberg et al. [34].

Physical activity levels, assessed using the APALQ (Table 2), increased from Pre-RHB to Post-RHB (7.94 ± 3.14 to 10.37 ± 3.50, mean difference = 2.4, 95% CI 1.8–3.0; *p* < 0.001) with a moderate effect size (Cohen’s d = 0.77). Regarding physical activity categories, the proportion of children classified as sedentary decreased from 77.39% to 49.57%, whereas those considered moderately active increased from 20.87% to 46.96%, and those classified as very active increased from 1.74% to 3.48%.

These improvements in APALQ were maintained throughout the 12M follow-up (Supplementary Figure S1). No significant changes were observed in body mass index (BMI) from Pre-RHB to Post-RHB (*p* = 0.920; Cohen’s d = −0.01).

In terms of peripheral muscle strength, significant changes were observed from Pre-RHB to Post-RHB (Table 2). Mean dominant HGS increased from 20.36 ± 8.72 kg at Pre-RHB to 22.77 ± 9.57 kg at Post-RHB (mean difference = 2.4 kg, 95% CI 1.4–3.4; *p* < 0.001; Cohen’s d = 0.46), while non-dominant HGS rose from 19.03 ± 8.60 kg at Pre-RHB to 20.73 ± 8.91 kg at Post-RHB (mean difference = 1.7 kg, 95% CI 0.68–2.7; *p* = 0.001; Cohen’s d = 0.32). When expressed as % predicted, both hands also showed significant changes (*p* < 0.001 for the dominant and *p* = 0.002 for the non-dominant hand). The dominant hand exhibited a slightly larger immediate increase at Post-RHB, whereas both sides followed a comparable pattern of slight decline and stabilisation at 6M and 12M follow-ups (Supplementary Figure S2).

Respiratory muscle strength, as reflected by PImax, showed a significant increase from 70.46 ± 25.98 cmH_2_O at Pre-RHB to 85.84 ± 29.27 cmH_2_O at Post-RHB (mean difference = 15 cmH_2_O, 95% CI 11–20; *p* < 0.001), with a large effect size (Cohen’s d = 0.92). Based on established paediatric reference values stratified by age and sex from Szeinberg et al., the proportion of participants below the lower limit of normal (LLN) decreased from 73.33% at Pre-RHB to 48.33% at Post-RHB, while those within the normal range rose from 11.67% to 38.33%. These changes were maintained at the 6M and 12M follow-ups (Supplementary Figure S3).

Ultrasound measures showed significant changes in rectus femoris muscle thickness (RF MT) (Table 2). Dominant-limb thickness rose from 12.88 ± 3.71 mm at Pre-RHB to 13.43 ± 3.75 mm at Post-RHB (mean difference = 0.56 mm, 95% CI 0.18–0.94; *p* = 0.005; Cohen’s d = 0.30), and non-dominant thickness from 12.25 ± 3.49 mm at Pre-RHB to 13.12 ± 3.59 mm at Post-RHB (mean difference = 0.87 mm, 95% CI 0.50–1.2; *p* < 0.001; Cohen’s d = 0.47). RF MT showed a gradual increase across follow-up assessments, with the dominant limb exhibiting slightly greater gains, and increases still evident at the 6M and 12M follow-ups (Supplementary Figure S4). Good to excellent reliability was observed for ultrasound measurements (ICC 0.82–0.91; Appendix A Table A1). Echo-intensity (RF EI) also showed significant changes from Pre-RHB to Post-RHB (*p* = 0.003), with abnormal Heckmatt grades (II–IV) declining from 20.83% to 9.38%.

Regarding fatigue, Peds FACIT-F scores increased from 25.00 ± 10.94 at Pre-RHB to 34.28 ± 12.48 at Post-RHB (mean difference = 9.3, 95% CI 7.1–11; *p* < 0.001), with a large effect size (Cohens’ d = 0.80), indicating reduced fatigue perception (higher scores denote better status). Category analysis showed a decrease in high/very high fatigue from 37.27% to 16.37%, while the fatigue-free category rose from 4.55% to 26.36%. These changes were observed at the 6M and 12M follow-ups (Supplementary Figure S5).

**Figure 3:**
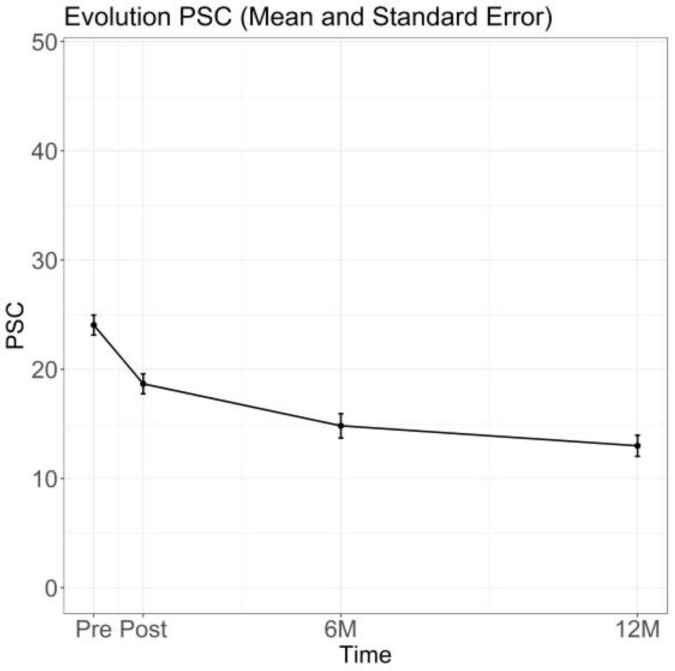
Evolution of PSC Performance Over Time. Values are shown as mean ± standard error at each assessment time point. Sample sizes at each time point are reported in Supplementary Table S7.

With respect to psychiatric symptoms, PSC scores decreased from 23.50 ± 9.18 at Pre-RHB to 18.99 ± 9.05 at Post-RHB (mean difference = −4.5, 95% CI −6.2 to −2.9; *p* < 0.001; Cohen’s d = −0.56). The proportion of paediatric participants with a positive screening for cognitive, emotional, and behavioural problems, declined from 25.00% to 15.62% at Post-RHB, with reductions remaining at the 12M follow-up (Figure 3). Detailed psychiatric diagnoses, timing of symptom detection, and pharmacological treatment among participants with a positive PSC screening are summarised in Appendix A Table A2.

**Figure 4:**
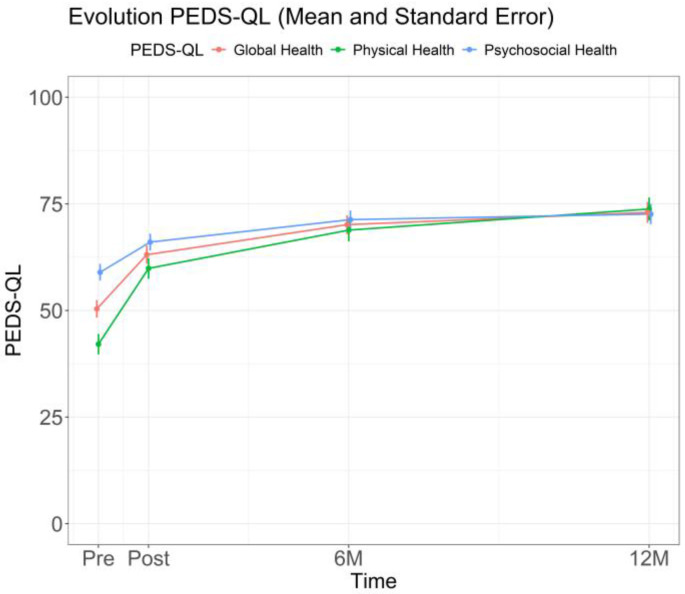
Evolution of PedsQL Performance Over Time. Values are shown as mean ± standard error at each assessment time point. Sample sizes at each time point are reported in Supplementary Table S8.

Finally, in terms of health-related quality of life, PedsQL scores showed significant changes from Pre-RHB to Post-RHB across all domains. Physical health showed the largest change, increasing from 42.56 ± 21.11 to 58.43 ± 23.39 (mean difference = 16, 95% CI 11–21; *p* < 0.001; Cohen’s d = 0.69). Psychosocial health rose from 58.67 ± 17.43 to 64.85 ± 18.29 (mean difference = 6.2, 95% CI 2.6–9.7; *p* < 0.001; Cohen’s d = 0.36), and global health from 50.40 ± 17.87 to 61.44 ± 19.44 (mean difference = 11, 95% CI 7.1–15; *p* < 0.001; Cohen’s d = 0.58). These changes were maintained at the 6M and 12M follow-ups (Figure 4).

## 4. Discussion

This quasi-experimental study is, to our knowledge, the first to investigate the association between a structured PR programme and clinical outcomes in PPCC patients. Over the five-week PR intervention, significant changes in exercise capacity were observed, alongside increases in inspiratory muscle strength and reductions in exertional dyspnoea and fatigue. Furthermore, significant changes were also observed across secondary outcomes, including physical function, quality of life, and psychiatric symptoms.

The management of PPCC remains primarily centred on symptom management with a gradual return to daily activities, and in this context, rehabilitation has emerged as a key component of multidisciplinary care. To date, most paediatric studies have focused on characterising persistent symptoms rather than exploring the potential benefits of therapeutic rehabilitation strategies [45]. Ashkenazy et al. assessed 90 children with PPCC in a multidisciplinary clinic but provided only descriptive accounts of symptoms, without systematic objective evaluations or structured rehabilitation protocols [46]. Garai et al. similarly reported persistent symptoms in 89 PPCC patients and included a quality of life questionnaire. They also mentioned supportive and individualised rehabilitation strategies; however, these approaches were not described, the number of patients receiving them was not reported, and their benefits were not assessed [47]. More recently, Ogonowska-Slodownik et al. conducted a randomised controlled trial in 40 children aged 10–12 years, reporting that an eight-week land- or water-based exercise training programme was associated with improvements in spirometric parameters. However, participants were not formally diagnosed with PPCC, as inclusion was based solely on post-infection fatigue or dyspnoea; those already engaged in regular exercise were excluded, and outcomes were restricted to lung function testing [48]. In contrast, our study systematically assessed a clinically defined PPCC population using validated physical, psychological, and quality of life measures, and implemented a structured PR programme with follow-up assessments to 12 months.

To date, no interventional studies have specifically evaluated PR in PPCC, limiting direct comparison with age-matched populations. In a recent systematic review and meta-analysis, Oliveira et al. [23] examined the effects of PR in adult patients with PCC. Relative to usual care without rehabilitation, PR was linked to statistically significant and clinically relevant gains in exercise capacity (assessed by 6MWT), as well as decreases in fatigue levels (measured by Fatigue Severity Scale (FSS)), whereas no statistically significant effects were observed for dyspnoea (measured by modified Medical Research Council dyspnoea scale (mMRC)) or peripheral muscle strength (measured by handgrip). Consistent with these findings, our study observed significant changes in exercise capacity and fatigue at Post-RHB, with moderate effect sizes. In contrast, we also observed changes in peripheral muscle strength and exertional dyspnoea. These differences may partly reflect variations in programme composition, as many studies included in the meta-analysis primarily emphasised aerobic training, whereas our intervention systematically incorporated both aerobic and strength training. Regarding dyspnoea, Oliveira et al. suggested that the absence of significant effects in adults may reflect low baseline dyspnoea scores, potentially resulting in a floor effect. In our study, perceived dyspnoea was assessed in relation to exertion, before and after the 6MWT, thereby capturing exertional dyspnoea, which represents one of the most common and functionally limiting symptoms in PPCC.

Exercise capacity was the primary outcome of this study. From Pre-RHB to Post-RHB, an increase of 37 metres in walking distance was observed, which may reflect improved tolerance to physical effort in a population frequently limited in daily activities by exertional symptoms. Although paediatric-specific minimal clinically important difference thresholds for the 6MWT are not well established, the magnitude of change observed in this study is comparable to improvements considered meaningful in other paediatric conditions, including neuromuscular disorders, where changes of approximately 30 metres have been proposed as clinically relevant [49].

At present, it remains unclear which type, intensity, or dose of physical activity is safe and beneficial for PPCC. In adults, several trials and systematic reviews have reported that exercise-based interventions are generally safe and feasible, although they require individual tailoring [50,51,52]. Nevertheless, despite evidence suggesting that PR in adults may be associated with reductions in fatigue in PCC, careful consideration is required when advising exercise-based interventions for individuals who report fatigue, to limit the risk of post-exertional symptom exacerbation and other undesirable effects [53]. In line with NICE guidance [54,55], our PPCC patients considered at risk of post-exertional symptom exacerbation were instructed to avoid exercising to the point of fatigue or symptom worsening, and graded exercise therapy was avoided. In this context, relevant changes in perceived fatigue were observed at Post-RHB, with the proportion of participants classified as having high or very high fatigue decreasing by more than half, from 37.27% at Pre-RHB to 16.37% at Post-RHB, while the proportion classified as fatigue-free increased markedly, from 4.55% to 26.36%.

Within this framework, inspiratory muscle training (IMT) has gained increasing attention in adults with PCC. Ortiz-Ortigosa et al. [56] concluded that the most effective exercise-based strategies combine aerobic and resistance training with IMT, while Morgan et al. reported beneficial effects of IMT on pulmonary function, dyspnoea, exercise capacity, and quality of life, including in home-based programmes [57]. In the present study, inspiratory muscle strength could only be assessed in a subset of participants (n = 57). During the early phase of the pandemic, systematic assessment was limited by the availability of appropriate single-use bacterial filters and adaptors required for safe respiratory testing. Within this subgroup, a high proportion of participants (73.53%) presented with reduced PImax at Pre-RHB, and this proportion decreased to 47.06% at Post-RHB. These changes were accompanied by significant increases in inspiratory muscle strength, with a large effect size, and by reductions in exertional dyspnoea assessed after the 6MWT. These findings should be interpreted with caution due to the limited sample size and the exploratory nature of the analysis; however, they are consistent with emerging evidence suggesting that inspiratory muscle weakness, probably reflecting diaphragmatic involvement, may play a role in the development of exertional dyspnoea in PPCC.

Physiological studies indicate that IMT may reduce neural respiratory drive and optimise breathing patterns, by improving the balance between respiratory muscle load and capacity. Langer et al. proposed that respiratory muscle hypertrophy induced by IMT, may attenuate both dyspnoea and diaphragmatic activation during exercise [58,59]. Taken together, these findings suggest that IMT, particularly when integrated into multicomponent rehabilitation programmes, may represent a relevant therapeutic component in the management of PPCC.

The mechanisms underlying PPCC are still not completely defined. In adults, persistent respiratory manifestations have been related to fibrotic alterations, whereas similar findings have not been reported in children [60]. Instead, our findings of inspiratory muscle weakness, in parallel with observed changes in dyspnoea, fatigue, and exercise capacity, are consistent with emerging hypotheses suggesting that persistent symptoms in PPCC may involve autonomic dysfunction of the vagus nerve, as has recently been proposed in adults with PCC [61].

Changes were also observed in peripheral muscle strength, as measured by HGS, and in quadriceps morphology, with increases in rectus femoris thickness and reductions in echo-intensity. Persistent physical symptoms in PPCC may substantially affect daily functioning and health-related quality of life, supporting the relevance of systematic assessment using instruments such as the PedsQL. From Pre-RHB to Post-RHB, changes in PedsQL scores were observed across physical, psychosocial, and global health domains, with the largest changes in physical health and maintenance of these changes at 6M and 12M follow-ups. Our findings are consistent with existing evidence indicating that multicomponent rehabilitation programmes, combining aerobic conditioning, strength training, and respiratory physiotherapy, are linked to larger gains in exercise capacity and health-related quality of life than unimodal therapeutic strategies [62].

Equally important, accumulating evidence indicates that children and adolescents with PPCC are at increased risk of developing mental health symptoms, including anxiety, depressive symptoms, and sleep disturbances [63,64]. This is particularly relevant given that childhood and adolescence represent critical developmental periods for the onset of psychiatric disorders, making this population especially vulnerable. Despite growing recognition of impaired mental health in Long COVID, data on the impact of rehabilitation interventions on psychological well-being in PPCC remain limited [65]. In our study sample, 25% of participants screened positive for cognitive, emotional, or behavioural problems at Pre-RHB, with a reduction observed at Post-RHB to 15.62%, which was maintained at the 12M follow-up. These changes were accompanied by higher school attendance and higher levels of physical activity. While causality cannot be inferred, these findings are in line with evidence suggesting that early detection of psychiatric symptoms and a multidisciplinary approach may facilitate timely intervention and may help prevent chronic impairment [66,67]. Taken together, our data suggest that a structured, multidisciplinary PR programme may support both physical and psychological domains of health in PPCC, consistent with a biopsychosocial model of care.

This study has several limitations. First, the quasi-experimental pre–post design, in the absence of a randomised control group, limits the ability to draw causal inferences. Although the inclusion of a non-intervention control group was considered unethical during the COVID-19 pandemic due to the absence of clinical equipoise, observed changes cannot be attributed exclusively to the PR programme. Spontaneous recovery over time, non-specific effects related to increased clinical attention, repeated assessments, and participant expectations may have contributed to the findings, which should therefore be interpreted with caution. Second, the single-centre design within a tertiary multidisciplinary unit may have led to a selection bias towards patients with more severe or persistent symptoms, or higher motivation for rehabilitation, limiting generalisability to broader paediatric populations. Third, adherence to the home-based component of the programme was not systematically monitored, and overall physical activity during the 12M follow-up could not be controlled, introducing uncertainty regarding the sustainability of observed changes. Fourth, PImax could only be assessed in a subset of participants due to logistical constraints during the early pandemic, when appropriate disposable bacterial filters and adaptors were unavailable, limiting the representativeness of inspiratory muscle findings. Finally, fatigue and quality of life outcomes relied on self-reported measures, which may be subject to response bias, particularly in younger participants. However, validated paediatric-specific instruments were used to enhance measurement reliability.

## 5. Conclusions

This quasi-experimental study contributes novel evidence suggesting that a structured pulmonary rehabilitation programme tailored to children and adolescents with PPCC is associated with significant changes in exercise capacity, inspiratory muscle strength, exertional dyspnoea and fatigue, as well as in peripheral muscle strength and muscle morphology. Beyond physical health, reductions in psychiatric symptoms and higher school attendance were also observed. These findings highlight the substantial burden of PPCC and underscore the need for targeted screening and individualised multidisciplinary care to support this vulnerable paediatric population.

In summary, multidisciplinary pulmonary rehabilitation may be associated with beneficial changes in physical health, psychological well-being, and overall quality of life in children and adolescents with PPCC. Future controlled studies are warranted to confirm these results and to optimise tailored rehabilitation programmes for paediatric populations.

## Data Availability

De-identified data supporting the findings of this study are available from the corresponding author upon reasonable request and subject to institutional and ethical approval.

## Supplementary Materials

The following supporting information can be downloaded at: https://www.mdpi.com/article/10.3390/jcm15020535/s1, Figure S1: Evolution of the Assessment of Physical Activity Levels Questionnaire (APALQ) over time.; Table S1: Background medical conditions of the study sample (N = 115).; Figure S2: Evolution of handgrip strength (HGS) over time.; Table S2: Sample size, mean values, and standard errors of the 6-min walk test (6MWT) at each assessment time point.; Figure S3: Evolution of maximal inspiratory pressure (PImax) over time.; Table S3: Sample size, mean values, and standard errors of the Assessment of Physical Activity Levels Questionnaire (APALQ) at each assessment time point.; Figure S4: Evolution of rectus femoris muscle thickness (RF MT) over time.; Table S4: Sample size, mean values, and standard errors of the handgrip strength (HGS) at each assessment time point.; Figure S5: Evolution of the Pediatric Functional Assessment of Chronic Illness Therapy-Fatigue (Peds FACIT-F) over time.; Table S5: Sample size, mean values, and standard errors of the maximal inspiratory pressure (PImax) at each assessment time point.; Table S6: Sample size, mean values, and standard errors of the rectus femoris muscle thickness (RF MT) at each assessment time point.; Table S7: Sample size, mean values, and standard errors of the Pediatric Symptom Checklist (PSC) at each assessment time point.; Table S8: Sample size, mean values, and standard errors of the Pediatric Quality of Life Inventory (PedsQL) at each assessment time point.; Table S9: Sample size, mean values, and standard errors of the Pediatric Functional Assessment of Chronic Illness Therapy-Fatigue (Peds FACIT-F) at each assessment time point.

## Appendix A

**Table A1:** Intraclass correlation coefficients (ICC) of rectus femoris muscle thickness ultrasound measurements (N = 115).

Variables	ICC Pre-RHB	ICC Post-RHB	ICC 6M Follow-Up	ICC 12M Follow-Up
RF MT (D), mm	0.867 [0.813; 0.906]	0.884 [0.836; 0.918]	0.898 [0.856; 0.928]	0.881 [0.833; 0.917]
RF MT (ND), mm	0.821 [0.752; 0.873]	0.91 [0.873; 0.937]	0.912 [0.875; 0.938]	0.882 [0.834; 0.917]

Abbreviations: RF MT, rectus femoris muscle thickness; D, dominant; ND, non-dominant; ICC, intraclass correlation coefficient; Pre-RHB, immediately before rehabilitation; Post-RHB, immediately after rehabilitation; 6M, 6 months after rehabilitation; 12M, 12 months after rehabilitation. Values are ICC with 95% CI based on two repeated measurements.

**Table A2:** Psychiatric diagnoses, timing of symptom detection, and pharmacological treatment in PPCC patients with positive PSC screening during follow-ups. (N = 44).

Variables	PPCC Patients
Diagnoses	
Trauma- and stressor-related disorders	14 (31.82)
Depressive disorders	9 (20.45)
Neurodevelopmental disorders	7 (15.91)
Anxiety disorders	5 (11.36)
No psychiatric diagnoses	4 (9.09)
Sleep–wake disorders	3 (6.82)
Feeding and eating disorders	1 (2.27)
ARMS	1 (2.27)
Number of diagnoses	1.00 (1.00; 1.50)
Diagnostic status	
New diagnoses	25 (56.82)
Previously known diagnoses	11 (25.00)
No psychopathology	8 (18.18)
Timing of psychiatric symptom detection	
Pre-RHB	35 (79.55)
Post-RHB	6 (13.64)
6M follow-up	2 (4.55)
Between Post-RHB and 6M follow-up	1 (2.27)
Pharmacological treatment	
No pharmacological treatment initiated	20 (45.45)
Pharmacological treatment initiated	19 (43.18)
Already on baseline pharmacological treatment	5 (11.36)

Abbreviations: PPCC, paediatric post-COVID-19 condition; Pre-RHB, immediately before rehabilitation; Post-RHB, immediately after rehabilitation; 6M, 6 months after rehabilitation; ARMS, At-Risk Mental State. Notes: Data are presented as median (Q1; Q3) or frequency (percentage).

## References

[1] World Health Organization (2023). A Clinical Case Definition of Post COVID-19 Condition in Children and Adolescents by a Delphi Consensus.

[2] Coughtrey A., Pereira S.M.P., Ladhani S., Shafran R., Stephenson T. (2025). Long COVID in children and young people: Then and now. Curr. Opin. Infect. Dis..

[3] Basaca D.-G., Jugănaru I., Belei O., Nicoară D.-M., Asproniu R., Stoicescu E.R., Mărginean O. (2025). Long COVID in children and adolescents: Mechanisms, symptoms, and long-term impact on health—A comprehensive review. J. Clin. Med..

[4] Luo D., Mei B., Wang P., Li X., Chen X., Wei G., Kuang F., Li B., Su S. (2024). Prevalence and risk factors for persistent symptoms after COVID-19: A systematic review and meta-analysis. Clin. Microbiol. Infect..

[5] Yang J., Markus K., Andersen K.M., Rudolph A.E., McGrath L.J., Nguyen J.L., Kyaw M.H., Whittle I., Blazos V., Heron L. (2024). Definition and measurement of post-COVID-19 conditions in real-world practice: A global systematic literature review. BMJ Open.

[6] Rao S., Gross R.S., Mohandas S., Stein C.R., Case A., Dreyer B., Pajor N.M., Bunnell H.T., Warburton D., Berg E. (2024). Postacute Sequelae of SARS-CoV-2 in Children. Pediatrics.

[7] Sansone F., Pellegrino G.M., Caronni A., Bonazza F., Vegni E., Lué A., Bocci T., Pipolo C., Giusti G., Di Filippo P. (2023). Long COVID in Children: A Multidisciplinary Review. Diagnostics.

[8] Bonilla H., Peluso M.J., Rodgers K., Aberg J.A., Patterson T.F., Tamburro R., Baizer L., Goldman J.D., Rouphael N., Deitchman A. (2023). Therapeutic trials for long COVID-19: A call to action from the interventions taskforce of the RECOVER initiative. Front. Immunol..

[9] Woo M.S., Shafiq M., Fitzek A., Dottermusch M., Altmeppen H., Mohammadi B., Mayer C., Bal L.C., Raich L., Matschke J. (2023). Vagus nerve inflammation contributes to dysautonomia in COVID-19. Acta Neuropathol..

[10] Bonaz B., Sinniger V., Pellissier S. (2020). Targeting the cholinergic anti-Inflammatory pathway with vagus nerve stimulation in patients with COVID-19?. Bioelectron. Med..

[11] Mazzone S.B., Undem B.J. (2016). Vagal Afferent Innervation of the Airways in Health and Disease. Physiol. Rev..

[12] Regmi B., Friedrich J., Jörn B., Senol M., Giannoni A., Boentert M., Daher A., Dreher M., Spiesshoefer J. (2023). Diaphragm Muscle Weakness Might Explain Exertional Dyspnea 15 Months after Hospitalization for COVID-19. Am. J. Respir. Crit. Care Med..

[13] Salem A.M., Al Khathlan N., Alogily M., Alharbi M., Alsubaei N., AlOuhali H., AlOtaibi A., Al Hamam A., Al Ghamdi K., Al-Asoom L. (2025). Respiratory muscle weakness, reduced exercise capacity, and impaired lung functions in long-term post-COVID-19 patients. Electron. J. Gen. Med..

[14] Spiesshoefer J., Regmi B., Senol M., Jörn B., Gorol O., Elfeturi M., Walterspacher S., Giannoni A., Kahles F., Gloeckl R. (2024). Potential Diaphragm Muscle Weakness-related Dyspnea Persists 2 Years after COVID-19 and Could Be Improved by Inspiratory Muscle Training: Results of an Observational and an Interventional Clinical Trial. Am. J. Respir. Crit. Care Med..

[15] Hopper S.I., Murray S.L., Ferrara L.R., Singleton J.K. (2019). Effectiveness of diaphragmatic breathing for reducing physiological and psychological stress in adults: A quantitative systematic review. JBI Evid. Synth..

[16] Bordoni B., Marelli F., Bordoni G. (2016). A review of analgesic and emotive breathing: A multidisciplinary approach. J. Multidiscip. Healthc..

[17] Zheng Y.-B., Zeng N., Yuan K., Tian S.-S., Yang Y.-B., Gao N., Chen X., Zhang A.-Y., Kondratiuk A.L., Shi P.-P. (2023). Prevalence and risk factor for long COVID in children and adolescents: A meta-analysis and systematic review. J. Infect. Public Health.

[18] Noij L., Terheggen-Lagro S., Muselaers E., Whittaker E., Gosling J., Brackel C., Oostrom K., Alsem M. (2024). A Multidisciplinary Approach: Management and Rehabilitation of Children with Pediatric Post-COVID-19 Condition. Pediatr. Infect. Dis. J..

[19] Saunders E.G., Pouliopoulou D.V., Miller E., Billias N., MacDermid J.C., Brunton L., Pereira T.V., Quinn K.L., Bobos P. (2025). Rehabilitation interventions and outcomes for post-COVID condition: A scoping review. BMJ Public Health.

[20] Li S., Dai B., Hou Y., Zhang L., Liu J., Hou H., Song D., Wang S., Li X., Zhao H. (2025). Effect of pulmonary rehabilitation for patients with long COVID-19: A systematic review and meta-analysis of randomized controlled trials. Ther. Adv. Respir. Dis..

[21] Pouliopoulou D.V., Macdermid J.C., Saunders E., Peters S., Brunton L., Miller E., Quinn K.L., Pereira T.V., Bobos P. (2023). Rehabilitation Interventions for Physical Capacity and Quality of Life in Adults With Post–COVID-19 Condition: A Systematic Review and Meta-Analysis. JAMA Netw. Open.

[22] Martínez-Pozas O., Meléndez-Oliva E., Rolando L.M., Rico J.A.Q., Corbellini C., Sánchez Romero E.A. (2024). The pulmonary rehabilitation effect on long COVID-19 syndrome: A systematic review and meta-analysis. Physiother. Res. Int..

[23] Oliveira M.R., Hoffman M., Jones A.W., Holland A.E., Borghi-Silva A. (2024). Effect of Pulmonary Rehabilitation on Exercise Capacity, Dyspnea, Fatigue, and Peripheral Muscle Strength in Patients With Post-COVID-19 Syndrome: A Systematic Review and Meta-Analysis. Arch. Phys. Med. Rehabil..

[24] Spruit M.A. (2014). Pulmonary rehabilitation. Eur. Respir. Rev..

[25] Barker K., Eickmeyer S. (2020). Therapeutic exercise. Med. Clin..

[26] Goicoechea-Calvo A., Coll-Fernández R., Navarro Expósito N., Colomer Giralt M., González-Aumatell A., Méndez-Hernández M., Carreras-Abad C., Pallarès Fontanet N., Tebe Cordomi C., Durà Mata M.J. (2025). Effects of Paediatric Post-COVID-19 Condition on Physical Function and Daily Functioning: A Cross-Sectional Study. Children.

[27] Gonzalez-Aumatell A., Bovo M.V., Carreras-Abad C., Cuso-Perez S., Domènech Marsal È., Coll-Fernández R., Goicoechea Calvo A., Giralt-López M., Enseñat Cantallops A., Moron-Lopez S. (2022). Social, Academic and Health Status Impact of Long COVID on Children and Young People: An Observational, Descriptive, and Longitudinal Cohort Study. Children.

[28] ATS Committee on Proficiency Standards for Clinical Pulmonary Function Laboratories (2002). ATS statement: Guidelines for the six-minute walk test. Am. J. Respir. Crit. Care Med..

[29] Borg G., Linderholm H. (1967). Perceived Exertion and Pulse Rate during Graded Exercise in Various Age Groups. Acta Med. Scand..

[30] MacDermid J., Solomon G., Valdes K. (2015). Clinical Assessment Recommendations.

[31] Serrano M.D.M., Collazos J.F.R., Romero S.M., Santurino M.S.M., Armesilla M.D.C., del Cerro J.L.P., de Espinosa M.G.-M. (2009). Dinamometría en niños y jóvenes de entre 6 y 18 años: Valores de referencia, asociación con tamaño y composición corporal. An. Pediatr..

[32] Gąsior J., Pawłowski M., Jeleń P., Rameckers E., Williams C., Makuch R., Werner B. (2020). Test–retest reliability of handgrip strength measurement in children and preadolescents. Int. J. Environ. Res. Public Health.

[33] American Thoracic Society/European Respiratory Society (2002). ATS/ERS Statement on respiratory muscle testing. Am. J. Respir. Crit. Care Med..

[34] Szeinberg A., Marcotte J.E., Roizin H., Mindorff C., England S., Tabachnik E., Levison H. (1987). Normal values of maximal inspiratory and expiratory pressures with a portable apparatus in children, adolescents, and young adults. Pediatr. Pulmonol..

[35] Agyapong-Badu S., Warner M., Samuel D., Narici M., Cooper C., Stokes M. (2014). Anterior thigh composition measured using ultrasound imaging to quantify relative thickness of muscle and non-contractile tissue: A potential biomarker for musculoskeletal health. Physiol. Meas..

[36] Heckmatt J.Z., Leeman S., Dubowitz V. (1982). Ultrasound imaging in the diagnosis of muscle disease. J. Pediatr..

[37] Martínez-Lemos I., Pérez C.A., Lastra A.S., Carral J.M.C., Sánchez R.V. (2016). Physical activity questionnaires for Spanish children and adolescents: A systematic review. An. Sist. Sanit. Navar..

[38] Zaragoza Casterad J., Generelo E., Aznar S., Abarca-Sos A., Julian J.A., Mota J. (2012). Validation of a short physical activity recall questionnaire completed by Spanish adolescents. Eur. J. Sport Sci..

[39] Rodríguez-Muguruza S., Ariza-Ariza R., Díaz del Campo P., Seoane-Mato D., Carmona L., García-Magariño M. (2024). Validation of FACIT-Fatigue in Spanish-speaking patients with rheumatoid arthritis. Rev. Colomb. Reumatol..

[40] Pérez-Ardanaz B., Morales-Asencio J.M., Peláez-Cantero M.J., García-Mayor S., Canca-Sánchez J.C., Martí-García C. (2022). Fatigue, quality of life, and use of healthcare resources in children with complex chronic diseases. An. Sist. Sanit. Navar..

[41] Lai J.-S., Cella D., Kupst M.J., Holm S., Kelly M.E., Bode R.K., Goldman S. (2007). Measuring fatigue for children with cancer: Development and validation of the pediatric Functional Assessment of Chronic Illness Therapy-Fatigue (pedsFACIT-F). J. Pediatr. Hematol. Oncol..

[42] Varni J.W., Seid M., Kurtin P.S. (2001). PedsQL™ 4.0: Reliability and validity of the Pediatric Quality of Life Inventory™ Version 4.0 generic core scales in healthy and patient populations. Med. Care.

[43] Ramírez S., Gana S., Godoy M.I., Valenzuela D., Araya R., Gaete J. (2023). Validation of the Spanish version of the Pediatric Symptom Checklist (PSC) to identify and assess psychosocial problems among early adolescents in Chile. PLoS ONE.

[44] von Elm E., Altman D.G., Egger M., Pocock S.J., Gøtzsche P.C., Vandenbroucke J.P. The Strengthening the Reporting of Observational Studies in Epidemiology (STROBE) Statement: Guidelines for Reporting Observational Studies. EQUATOR Network. https://www.equator-network.org/reporting-guidelines/strobe/.

[45] Berg S.K., Nielsen S.D., Nygaard U., Bundgaard H., Palm P., Rotvig C., Christensen A.V. (2022). Long COVID symptoms in SARS-CoV-2-positive adolescents and matched controls (LongCOVIDKidsDK): A national, cross-sectional study. Lancet Child Adolesc. Health.

[46] Ashkenazi-Hoffnung L., Shmueli E., Ehrlich S., Ziv A., Bar-On O., Birk E., Lowenthal A., Prais D. (2021). Long COVID in Children: Observations From a Designated Pediatric Clinic. Pediatr. Infect. Dis. J..

[47] Garai R., Krivácsy P., Herczeg V., Kovács F., Tél B., Kelemen J., Máthé A., Zsáry E., Takács J., Veres D.S. (2022). Clinical assessment of children with long COVID syndrome. Pediatr. Res..

[48] Ogonowska-Slodownik A., Labecka M.K., Maciejewska-Skrendo A., Morgulec-Adamowicz N., Starczewski M., Gajewski J., McNamara R.J., Kaczmarczyk K. (2025). Effect of water- and land-based exercise on lung function in children with post-COVID-19 condition: Secondary results from a randomised controlled trial. ERJ Open Res..

[49] McDonald C.M., Henricson E.K., Han J.J., Abresch R.T., Nicorici A., Elfring G.L., Atkinson L., Reha A., Miller L.L. (2010). The 6-minute walk test as a new outcome measure in Duchenne muscular dystrophy. Muscle Nerve.

[50] Jimeno-Almazán A., Franco-López F., Buendía-Romero Á., Martínez-Cava A., Sánchez-Agar J.A., Sánchez-Alcaraz Martínez B.J., Courel-Ibáñez J., Pallarés J.G. (2022). Rehabilitation for post-COVID-19 condition through a supervised exercise intervention: A randomized controlled trial. Scan. J. Med. Sci. Sports.

[51] Fernández-Lázaro D., Santamaría G., Sánchez-Serrano N., Lantarón Caeiro E., Seco-Calvo J. (2022). Efficacy of Therapeutic Exercise in Reversing Decreased Strength, Impaired Respiratory Function, Decreased Physical Fitness, and Decreased Quality of Life Caused by the Post-COVID-19 Syndrome. Viruses.

[52] Barker-Davies R.M., Oliver O., Senaratne K.P.P., Baker P., Cranley M., Dharm-Datta S., Ellis H., Goodall D., Gough M., Lewis S. (2020). The Stanford Hall consensus statement for post-COVID-19 rehabilitation. Br. J. Sports Med..

[53] Larun L., Brurberg K.G., Odgaard-Jensen J., Price J.R. (2017). Exercise Therapy for Chronic Fatigue Syndrome. Cochrane Database Syst. Rev..

[54] National Institute for Health and Care Excellence (NICE) (2020). COVID-19 Rapid Guideline: Managing the Long-Term Effects of COVID-19.

[55] National Institute for Health Research (NIHR) (2021). Living with COVID-19—Second Review.

[56] Ortiz-Ortigosa L., Gálvez-Álvarez P., Viñolo-Gil M.J., Rodríguez-Huguet M., Góngora-Rodríguez J., Martín-Valero R. (2024). Effectiveness of pulmonary rehabilitation programmes and/or respiratory muscle training in patients with post-COVID conditions: A systematic review. Respir. Res..

[57] Morgan S.P., Visovsky C., Thomas B., Klein A.B. (2023). Respiratory Muscle Strength Training in Patients Post-COVID-19: A Systematic Review. Clin. Nurs. Res..

[58] Langer D., Ciavaglia C., Faisal A., Webb K.A., Neder J.A., Gosselink R., Dacha S., Topalovic M., Ivanova A., O’Donnell D.E. (2018). Inspiratory muscle training reduces diaphragm activation and dyspnea during exercise in COPD. J. Appl. Physiol..

[59] Charususin N., Dacha S., Gosselink R., Decramer M., Von Leupoldt A., Reijnders T., Louvaris Z., Langer D. (2018). Respiratory muscle function and exercise limitation in patients with chronic obstructive pulmonary disease: A review. Expert Rev. Respir. Med..

[60] Bakhtiari E., Moazzen N. (2024). Pulmonary function in children post-SARS-CoV-2 infection: A systematic review and meta-analysis. BMC Pediatr..

[61] Lladós G., Massanella M., Coll-Fernández R., Rodríguez R., Hernández E., Lucente G., López C., Loste C., Santos J.R., España-Cueto S. (2024). Vagus nerve dysfunction in the post-COVID-19 condition: A pilot cross-sectional study. Clin. Microbiol. Infect..

[62] Presta V., Guarnieri A., Laurenti F., Mazzei S., di Martino O., Vitale M., Condello G. (2025). Post-Acute COVID-19 Syndrome (PACS) and Exercise Interventions: A Systematic Review of Randomized Controlled Trials. Sports.

[63] Oh J., Kang J., Yon D.K. (2025). Editorial: Mental Health Distress in Long COVID Condition Among the Pediatric Population: A Contemporary Medical Challenge. J. Am. Acad. Child Adolesc. Psychiatry.

[64] Taquet M., Sillett R., Zhu L., Mendel J., Camplisson I., Dercon Q., Harrison P.J. (2022). Neurological and psychiatric risk trajectories after SARS-CoV-2 infection: An analysis of 2-year retrospective cohort studies including 1,284,437 patients. Lancet Psychiatry.

[65] Kubota T., Kuroda N., Sone D. (2023). Neuropsychiatric aspects of long COVID: A comprehensive review. Psychiatry Clin. Neurosci..

[66] Stephenson T., Pereira S.M.P., Shafran R., De Stavola B.L., Rojas N., McOwat K., Simmons R., Zavala M., O’mahoney L., Chalder T. (2022). Physical and mental health 3 months after SARS-CoV-2 infection (long COVID) among adolescents in England (CLoCk): A national matched cohort study. Lancet Child Adolesc. Health.

[67] Moore D.A., Nunns M., Shaw L., Rogers M., Walker E., Ford T., Garside R., Ukoumunne O., Titman P., Shafran R. (2019). Interventions to improve the mental health of children and young people with long-term physical conditions: Linked evidence syntheses. Health Technol. Assess..

