# Supplementary material for: Impact of Pulmonary Rehabilitation on Physical, Mental Health and Quality of Life in Children with Post-COVID-19 Condition: A 12-Month Quasi-Experimental Study
Source: J Clin Med. 2026 Jan 9;15(2):535. doi: 10.3390/jcm15020535 (PMC12842031; doi:10.3390/jcm15020535)
Supplement: Supplementary file 1 [file jcm-15-00535-s001.zip › jcm-4013246-supplementary.pdf]

Supplementary Tables

Supplementary Table S1. Background medical conditions of the study sample (N = 115).

	**PPCC patients N = 115**
Respiratory system disorders	
Asthma	12 (10.43)
Bronchitis	11 (9.57)
Recurrent pneumonia in childhood	2 (1.74)
Bronchiolitis in childhood	3 (2.61)
Cardiovascular disorders	
Wolff Parkinson white syndrome[b]	1 (0.87)
Interatrial communication	1 (0.87)
Migraine	9 (7.83)
Postinfectious cerebellitis[b]	1 (0.87)
Duane syndrome	1 (0.87)
Recurrent nonspecific headache	8 (6.96)
Dyslexia	4 (3.48)
Autism spectrum disorder (level 1)	3 (2.61)
Psychiatric disorders	
Depression	5 (4.35)
Attention deficit hyperactivity disorder	9 (7.83)
Anxiety	5 (4.35)
Adjustment disorder	1 (0.87)
Autoimmune system disorders	
Lupus erythematosus	1 (0.87)
Autoimmune hypothyroidism	2 (1.74)
Celiac disease	3 (2.61)
Traumatological disorders	
Osgood Schlatter disease	1 (0.87)
Scoliosis	3 (2.61)
Sever's disease[b]	1 (0.87)
Otovestibular disorders	
Peripheral vertigo	2 (1.74)
Labyrinthitis	1 (0.87)
Conductive hearing loss	2 (1.74)
Endocrine–metabolic disorders	
Obesity	2 (1.74)
Subclinical hypothyroidism	1 (0.87)
Short stature[b]	2 (1.74)
Gastrointestinal disorders	
Helicobacter pylori infection[b]	2 (1.74)
Mesenteric adenitis	1 (0.87)
Allergic disorders	
Atopic dermatitis	22 (19.13)
Rhinoconjunctivitis	10 (8.70)
Chronic urticaria	2 (1.74)
Other Disorders	
Chronic fatigue	1 (0.87)
Heterozygous mutation of factor V Leiden	1 (0.87)
Haemochromatosis	1 (0.87)
Chromosome 3q29 microdeletion	1 (0.87)
Polydactyly	1 (0.87)
Vitiligo	1 (0.87)
Dengue[b]	1 (0.87)

Hereditary angioedema	1 (0.87)

Abbreviations: PPCC, paediatric post-COVID-19 condition. Notes: [b] These medical conditions were resolved during the present study. Data are presented as frequency (percentage).

Supplementary Table S2. Sample size, mean values, and standard errors of the 6-minute walk test (6MWT) at each assessment time point.

6MWT	Pre-RHB	Post-RHB	6M follow-up	12M follow-up
N	110	106	86	85
Mean	509	543.26	568.41	581.36
Standard error	8.21	8.67	9.17	8.16

Abbreviations: Pre-RHB, immediately before rehabilitation; Post-RHB, immediately after rehabilitation; 6M, 6 months after rehabilitation; 12M, 12 months after rehabilitation; 6MWT, 6-minute walk test.

Supplementary Table S3. Sample size, mean values, and standard errors of the Assessment of Physical Activity Levels Questionnaire (APALQ) at each assessment time point.

APALQ	Pre-RHB	Post-RHB	6M follow-up	12M follow-up
N	115	115	90	90
Mean	7.94	10.37	10.98	11.46
Standard error	0.29	0.33	0.45	0.47

Abbreviations: Pre-RHB, immediately before rehabilitation; Post-RHB, immediately after rehabilitation; 6M, 6 months after rehabilitation; 12M, 12 months after rehabilitation; APALQ, Assessment of Physical Activity Levels Questionnaire.

Supplementary Table S4. Sample size, mean values, and standard errors of the handgrip strength (HGS) at each assessment time point.

HGS (D)	Pre-RHB	Post-RHB	6M follow-up	12M follow-up
N	113	108	86	85
Mean	20.08	22.63	21.55	21.03
Standard error	0.81	0.93	0.99	0.94
**HGS (ND)**	**Pre-RHB**	**Post-RHB**	**6M follow-up**	**12M follow-up**
N	113	108	87	85
Mean	18.83	20.57	20.43	19.44
Standard error	0.80	0.87	1.00	0.93

Abbreviations: Pre-RHB, immediately before rehabilitation; Post-RHB, immediately after rehabilitation; 6M, 6 months after rehabilitation; 12M, 12 months after rehabilitation; D, dominant; ND, non-dominant; HGS, handgrip strength.

Supplementary Table S5. Sample size, mean values, and standard errors of the maximal inspiratory pressure (PImax) at each assessment time point.

PImax	Pre-RHB	Post-RHB	6M follow-up	12M follow-up
**N**	65	65	49	50
**Mean**	68.71	84.23	86.14	90.18
**Standard error**	3.25	3.67	3.58	3.76

Abbreviations: Pre-RHB, immediately before rehabilitation; Post-RHB, immediately after rehabilitation; 6M, 6 months after rehabilitation; 12M, 12 months after rehabilitation; PImax, maximal inspiratory pressure.

Supplementary Table S6. Sample size, mean values, and standard errors of the rectus femoris muscle thickness (RF MT) at each assessment time point.

RF MT (D)	Pre-RHB	Post-RHB	6M follow-up	12M follow-up
**N**	106	100	82	80
**Mean**	12.67	13.36	14.17	14.17
**Standard error**	0.36	0.37	0.38	0.39
**RF MT (ND)**	**Pre-RHB**	**Post-RHB**	**6M follow-up**	**12M follow-up**
**N**	106	100	82	80
**Mean**	12.12	13.05	13.84	14.25
**Standard error**	0.33	0.36	0.38	0.40

Abbreviations: Pre-RHB, immediately before rehabilitation; Post-RHB, immediately after rehabilitation; 6M, 6 months after rehabilitation; 12M, 12 months after rehabilitation; D, dominant; ND, non-dominant; RF MT, rectus femoris muscle thickness.

Supplementary Table S7. Sample size, mean values, and standard errors of the Pediatric Symptom Checklist (PSC) at each assessment time point.

PSC	Pre-RHB	Post-RHB	6M follow-up	12M follow-up
**N**	109	98	58	56
**Mean**	24.06	18.67	14.83	13.00
**Standard error**	0.92	0.92	1.12	0.97

Abbreviations: Pre-RHB, immediately before rehabilitation; Post-RHB, immediately after rehabilitation; 6M, 6 months after rehabilitation; 12M, 12 months after rehabilitation; PSC, Pediatric Symptom Checklist.

Supplementary Table S8. Sample size, mean values, and standard errors of the Pediatric Quality of Life Inventory (PedsQL) at each assessment time point.

PedsQL Global Health	Pre-RHB	Post-RHB	6M follow-up	12M follow-up
**N**	95	107	82	81
**Mean**	50.39	63.09	70.12	72.99
**Standard error**	1.85	1.90	2.00	2.24
**PedsQL Physical Health**	**Pre-RHB**	**Post-RHB**	**6M follow-up**	**12M follow-up**
**N**	98	110	83	82
**Mean**	42.10	59.87	68.86	73.81
**Standard error**	2.17	2.24	2.24	2.50
**PedsQL Psychosocial Health**	**Pre-RHB**	**Post-RHB**	**6M follow-up**	**12M follow-up**
**N**	98	110	83	82
**Mean**	59.98	66.05	71.32	72.63
**Standard error**	1.76	1.77	1.91	2.18

Abbreviations: Pre-RHB, immediately before rehabilitation; Post-RHB, immediately after rehabilitation; 6M, 6 months after rehabilitation; 12M, 12 months after rehabilitation; PedsQL, Pediatric Quality of Life Inventory.

Supplementary Table S9. Sample size, mean values, and standard errors of the Pediatric Functional Assessment of Chronic Illness Therapy-Fatigue (Peds FACIT-F) at each assessment time point.

Peds FACIT-F	Pre-RHB	Post-RHB	6M follow-up	12M follow-up
**N**	115	110	84	82
**Mean**	24.51	34.28	38.27	39.39
**Standard error**	1.03	1.19	1.12	1.36

Abbreviations: Pre-RHB, immediately before rehabilitation; Post-RHB, immediately after rehabilitation; 6M, 6 months after rehabilitation; 12M, 12 months after rehabilitation; Peds FACIT-F, Pediatric Functional Assessment of Chronic Illness Therapy-Fatigue.

Supplementary Figures

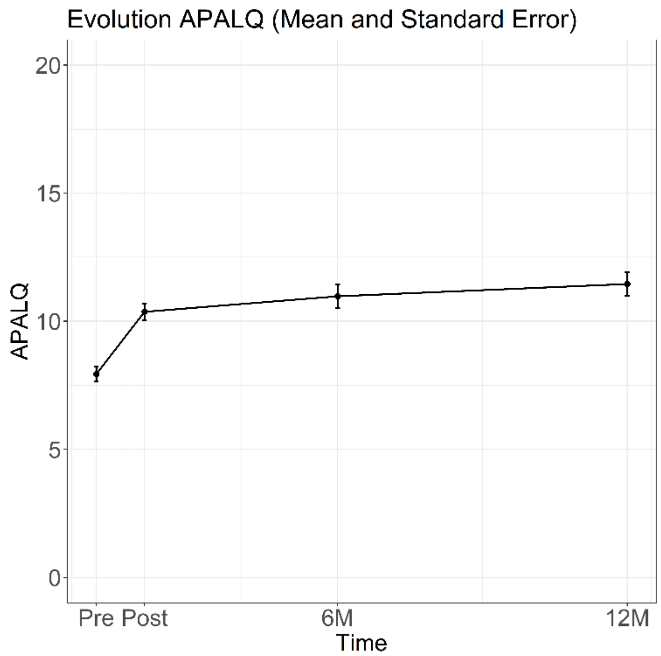

Supplementary Figure S1. Evolution of the Assessment of Physical Activity Levels Questionnaire (APALQ) over time. Values are shown as mean ± standard error at each assessment time point. Sample sizes at each time point are reported in Supplementary Table A3.

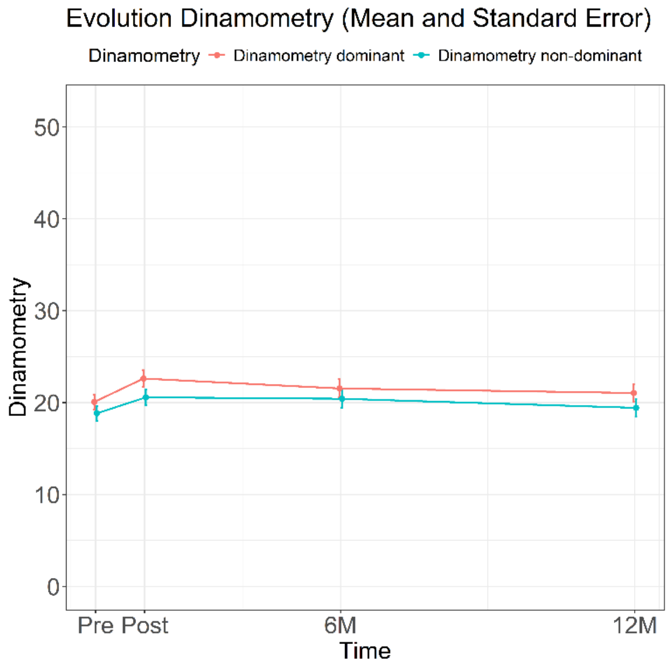

Supplementary Figure S2. Evolution of handgrip strength (HGS) over time. Values are shown as mean ± standard error at each assessment time point. Sample sizes at each time point are reported in Supplementary Table A4.

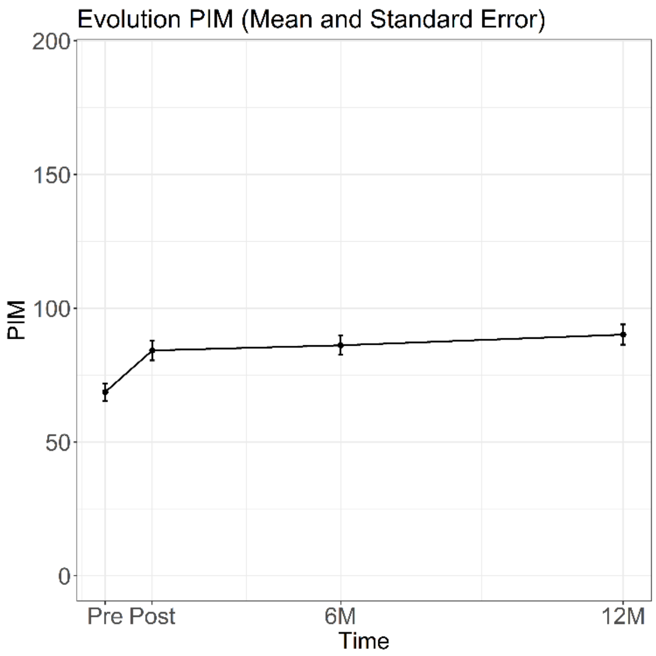

Supplementary Figure S3. Evolution of maximal inspiratory pressure (PImax) over time. Values are shown as mean ± standard error at each assessment time point. Sample sizes at each time point are reported in Supplementary Table A5.

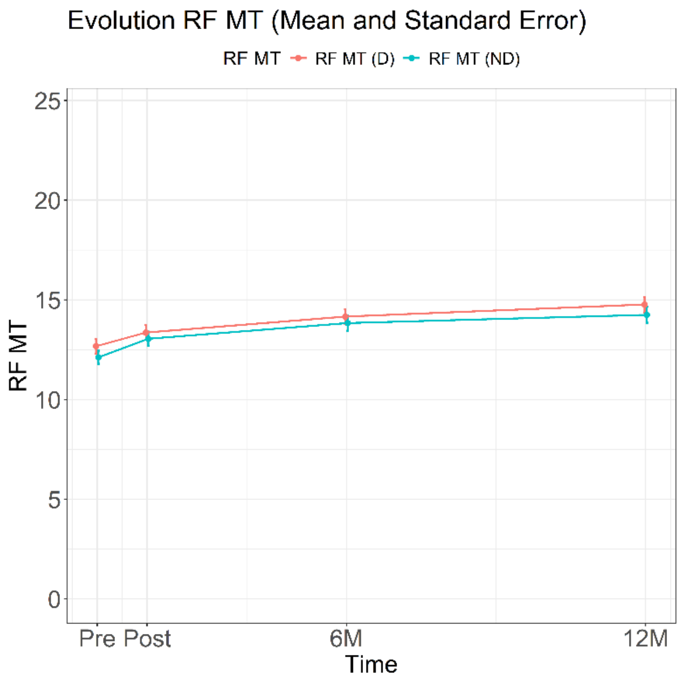

Supplementary Figure S4. Evolution of rectus femoris muscle thickness (RF MT) over time. Values are shown as mean ± standard error at each assessment time point. Sample sizes at each time point are reported in Supplementary Table A6.

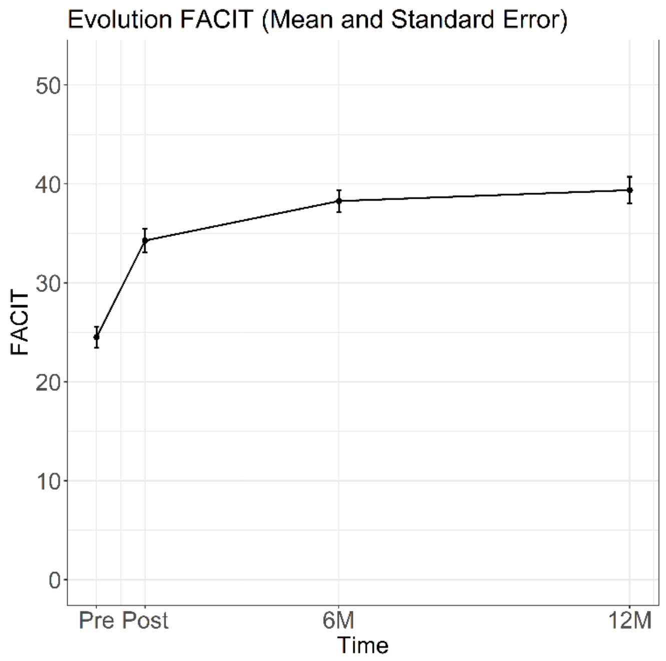

Supplementary Figure S5. Evolution of the Pediatric Functional Assessment of Chronic Illness Therapy-Fatigue (Peds FACIT-F) over time. Values are shown as mean ± standard error at each assessment time point. Sample sizes at each time point are reported in Supplementary Table A9.
